# Transcriptome-Powered Pluripotent Stem Cell Differentiation for Regenerative Medicine

**DOI:** 10.3390/cells12101442

**Published:** 2023-05-22

**Authors:** Derek A. Ogi, Sha Jin

**Affiliations:** 1 Department of Biomedical Engineering, Thomas J. Watson College of Engineering and Applied Sciences, State University of New York at Binghamton, Binghamton, NY 13902, USA; dogi1@binghamton.edu; 2 Center of Biomanufacturing for Regenerative Medicine, State University of New York at Binghamton, Binghamton, NY 13902, USA

**Keywords:** transcriptomics, human stem cell differentiation, RNA sequencing and data analysis, differential gene expression

## Abstract

Pluripotent stem cells are endless sources for in vitro engineering human tissues for regenerative medicine. Extensive studies have demonstrated that transcription factors are the key to stem cell lineage commitment and differentiation efficacy. As the transcription factor profile varies depending on the cell type, global transcriptome analysis through RNA sequencing (RNAseq) has been a powerful tool for measuring and characterizing the success of stem cell differentiation. RNAseq has been utilized to comprehend how gene expression changes as cells differentiate and provide a guide to inducing cellular differentiation based on promoting the expression of specific genes. It has also been utilized to determine the specific cell type. This review highlights RNAseq techniques, tools for RNAseq data interpretation, RNAseq data analytic methods and their utilities, and transcriptomics-enabled human stem cell differentiation. In addition, the review outlines the potential benefits of the transcriptomics-aided discovery of intrinsic factors influencing stem cell lineage commitment, transcriptomics applied to disease physiology studies using patients’ induced pluripotent stem cell (iPSC)-derived cells for regenerative medicine, and the future outlook on the technology and its implementation.

## 1. Introduction

Stem cells are able to differentiate into different cell types depending on their potency. The two most utilized sources of human pluripotent stem cells (hPSCs) are human embryonic stem cells (hESCs) and induced pluripotent stem cells (iPSCs). They are utilized in organ regeneration, cell and tissue transplantation, drug testing, tissue repair, wound healing, and diverse disease treatments after induced lineage specification. Over the past two decades, tremendous efforts have been made to generate favorable tissues or cell types through in vitro stem cell differentiation with the proper extracellular conditions. Cell–cell and cell–microenvironment interactions have a large impact on stem cell differentiation [1]. Extracellular matrix (ECM) compositions vary depending on the tissue type, which may create better microenvironmental conditions to guide hPSC differentiation into specific cell types and organoids [1,2,3,4]. In addition, the regulation of cellular transcriptome expression plays an important role in stem cell lineage commitment [5,6]. Studies in this regard have focused on understanding the complex interplay between gene expression and cell fate as cells specify distinct lineages. Microfabricated substrates provided optimal topographical placement for cell–cell interactions, promoting cell signaling for the lineage commitment of stem cells [7,8,9]. Growth factor nanoparticle delivery has been implicated in osteogenic differentiation, and laminin expression promotes differentiation into many cell types, including cardiomyocytes, keratinocytes, and retinal cells [9,10]. However, the generation of biologically mature cells/tissues from hPSCs has been challenging due to the complexity of stepwise developmental trajectories during differentiation and the multiple extrinsic and intrinsic regulatory components. In the process of hPSC differentiation, transcription factors are the most critical intrinsic regulators in cell fate decisions [11]. For example, *FLK2* and *THY1.1* promote the lineage specification of hematopoietic stem cells (HSCs) toward either short-term HSCs, long-term HSCs, or a multipotent progenitor population, depending on the expression levels of the two genes [12]. Short- and long-term HSCs are retained in the bone marrow and are characterized by the duration that they will remain as HSCs. The characterized multipotent progenitor populations are related to either myeloid progenitor cells or the more specified myeloid-committed oligopotent progenitor cells [12]. THY1.1lo− and FLK2− cells committed to a long-term HSC cell fate, THY1.1lo+ and FLK2+ cells differentiated into short-term HSCs, and the multipotent progenitor population was characterized by THY1.1− and FLK2+ expression [12].

One approach to analyzing global transcriptome expression, including transcription factors, is through RNA sequencing (RNAseq) and subsequent data analysis. RNAseq technologies generate the transcriptomic data of individual cells and allow the determination of how gene expression varies through the comparison of differential gene expression (DGE) among biological samples [13,14]. They generate high-quality genomic information for single cells or groups of cells. Hence, they have been widely applied to characterize the heterogeneity of hPSC-derived cells and cellular specification. Determining marker genes for each cell type allows for the more accurate cell typing of individual cells. Promoting the expression of these genes could also lead to increased differentiation toward that specific fate. Furthermore, DGE analysis offers an indicative assessment of the status of hPSC differentiation toward a specific cell or tissue type and the maturity of the hPSC-derived cells and organoids. Due to high heterogeneity within tissues, it is important to screen differentiated cells individually. Hence, single-cell RNA sequencing (scRNAseq) has been utilized for high-throughput RNAseq data analysis to assess each individual cell’s biological identity [15]. This review focuses on RNAseq techniques, tools for RNAseq data interpretation, RNAseq data analytic methods and their utilities, and transcriptomics-enabled human stem cell differentiation. In addition, the review outlines the potential benefits of the transcriptomics-aided discovery of intrinsic factors influencing stem cell lineage commitment, transcriptomics for disease physiology studies using patients’ iPSC-derived cells, and the future outlook on the technology and its implementation.

## 2. RNAseq Techniques

RNAseq provides the transcriptomic information that gives a cell its own identity. scRNAseq allows for gene expression analysis and interpretation at as low as the cellular level [16]. It is performed by first extracting the RNA, reverse transcribing RNA to a cDNA library, scaling up the generated cDNA library, and then sequencing the library [13,17,18,19]. Although the reverse transcription of RNA into cDNA might introduce some errors that limit the accuracy of sequence quantification [20], the accuracy of RNAseq of bulk cells is comparable to that of traditional quantitative reverse transcription PCR (qRT-PCR) in both the gene expression level and transcriptome complexity [21]. Furthermore, single-nucleus RNA sequencing (sNuc-Seq) that uses isolated nuclei is available with comparable transcriptome analysis sensitivity and cell type classification ability [22]. Several different single-cell and sNuc-Seq methods are utilized for sequencing. Table 1 highlights the methods of different RNAseq techniques, their advantages, and their limitations. The RNAseq methods can be either low throughput, such as Smart-seq2 and CEL-Seq2, or high throughput, such as Drop-seq, Seq-Well, or sci-RNA-seq [22]. RNAseq has also been accomplished by focusing specifically on the 5′-end with different methods, such as CAGE, RAMPAGE, STRT, NanoCAGE XL, and Oligo-capping [23]. All methods provide useful data but vary from one another in data consistency, cost, sensitivity, and duration [22]. sNuc-Seq is utilized for the sequencing of cells that are not easily separated into single cells, such as those in the brain, muscle, and adipose tissue [22]. There are associated limitations with sNuc-Seq since it does not consider transcriptomic data outside of the nucleus like scRNAseq or bulk RNAseq [22]. Bulk RNAseq differs from scRNAseq in that it measures the average gene expression of many cells rather than an individual cell’s genome expression. Thus, bulk RNAseq provides less data variability between samples compared to scRNAseq or sNuc-Seq sequencing [22] and is unable to distinguish unique cell populations in biological samples [24]. This limitation results from the inability to separate cell types in heterogeneous tissues so that the average genome expression from bulk sequencing cannot determine each cell type’s unique genome expression.

**Figure 1:**
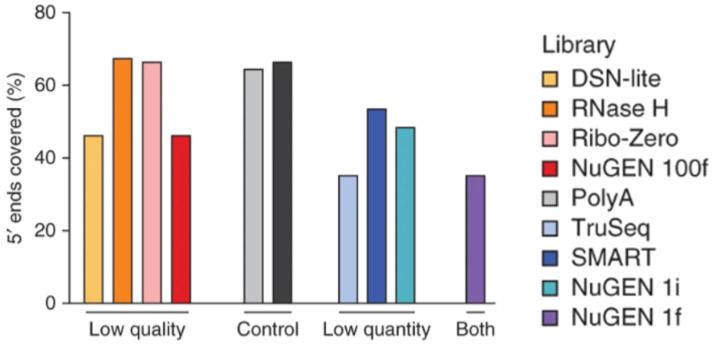
The 5′-to-3′ sequence coverage of low-quantity and low-quality samples utilizing different RNAseq analysis techniques. Reproduced with permission from Adiconis, Nature Methods; published by Springer Nature, 2013 [34].

Interestingly, certain RNAseq methods, such as Ribo-Zero, RNase H, duplex-specific nuclease, NuGEN, and SMART, have been developed for low-input and degraded samples with varying success [34]. The RNase H sequencing method was the most successful out of the observed methods in obtaining high values for the number of genes detected, the percentages of 5′ and 3′ covered, and Pearson correlation coefficients, with the lowest levels of rRNA and coefficient of variation [34]. The comparison of 5′-to-3′ sequence coverage for the observed low-input methods is shown in Figure 1. The Ribo-Zero sequencing method was also effective in transcribing moderately degraded samples with comparable 5′-to-3′ coverage [34,35]. However, Ribo-Zero loses its effectiveness if the RNA input is extremely degraded. In contrast, another method known as RNA Access RNAseq remains effective even for severely degraded samples as long as the input sample is larger than 5 nanograms [35]. However, many sequencing methods are inadequate to use for these samples due to poor observed performance and technical drawbacks [34]. These inadequate RNAseq methods utilize oligo(dT) to isolate RNA, particularly poly(A)^+^ RNA [34]. These methods could not generate significant results given low-quality or low-quantity readings due to a failure to observe poly(A)− or ribosomal RNA transcripts. Nevertheless, oligo(dT) is utilized as a primer for the reverse transcription of RNA into cDNA for most high-throughput scRNAseq methods, including 5′ capture, Drop-seq, Seq-well, sci-RNA-seq, 10x, and inDrop [36,37]. It is also utilized for a few low-throughput scRNAseq methods, such as Smart-seq and CEL-seq, and bulk sequencing methods, such as TagSeq, QuantSeq, and 3′ Pool-seq [37]. While oligo(dT)-related RNAseq methods are not optimal for degraded sample sequencing, methods such as RNase H, Ribo-Zero, NuGEN, and SMART can more effectively transcribe limited transcriptomic information [34].

Additionally, paired-end sequencing was suggested to provide substantial benefits to the sequencing yield compared to single-end sequencing [38]. Single-end sequencing involves only sequencing a single side of the cDNA generated during RNAseq, whereas paired-end sequencing involves sequencing both ends of the cDNA [38]. Inclusion and exclusion isoforms can be better detected in paired-end sequencing compared to single-end sequencing due to the increased ability to detect alternative exons [38]. Paired-end sequencing is also more cost-effective compared to single-end sequencing, especially when using short paired-end reads compared to long single-end reads [39]. Further, paired-end reads provide better gene expression results for each isoform and more characterized DGE results compared to single-end reads [39].

## 3. Tools for RNAseq Data Interpretation

There are several data analysis tools for interpreting RNAseq results applied to hPSC differentiation into diverse cell types. Each software tool interprets the data in a slightly different way, but each generates meaningful data. These different analysis packages and their uses, alongside the software that runs each program, are displayed in Table 2. These data include information on the DGE of the cells and can also include more specified information on cell clustering, cell subpopulation identification, network reconstruction, pseudotime analysis, and how transcriptomic expression changes given varying environmental or cellular conditions [40]. Pseudotime analysis is the process of developing a continuous trajectory for the genome expression of the cell over a specified time course [41]. Saturation, or the determination that sufficient sequencing has occurred for a specific sample, can be analyzed through software tools such as vegan [42]. Three analysis methods utilized for DGE are limma, DESeq2, and EdgeR [43]. They have differences in the approach to processing the data, for which limma utilizes a linear model, while DESeq2 and EdgeR use a negative binomial distribution [43]. The normalization of RNA data is not necessary for DESeq2, while the other two methods require it. DESeq2 also utilizes local regression to create bounds for the mean and variance of the data, as well as utilizes the Bayes theorem to generate gene movement and apply thresholds for gene expression based on available information [44]. EdgeR and the similar baySeq analysis method use the Bayesian empirical method to moderate transcript overdispersion [44]. The limma package is recommended to be used alongside other analysis methods, such as the EdgeR-associated package voom, which is used to normalize the data [44]. Myrna is another software analysis tool that determines the DGE from RNAseq data and is particularly useful for larger datasets [45]. Myrna utilizes parametric and non-parametric permutation testing to determine DGE after gene or exon coverage is calculated post-alignment [45]. Not all sequencing analysis methods are focused on DGE. MAP-RSeq is software focused on the generation of transcriptome alignment, gene and exon counts, fusion transcript information, and single-nucleotide variant information [46]. This software is intended to generate information on transcript quantification and should be paired with other downstream software such as EdgeR to determine DGE [46]. Variations in the datasets make certain analysis methods significantly more accurate depending on the environmental conditions of the experiment. EBSeq, which is similar in function to the baySeq analysis method, is beneficial for identifying differentially expressed isoforms, whereas EdgeR is better for determining biological variation [44]. NOIseq is a method that determines the model’s noise but is limited in that replication is not possible. Other analysis software such as sleuth is more beneficial for filtering and observing transcripts that occur in low abundance [44]. Based on the information needed for a specific dataset, different software packages can be used to obtain the relevant information. Software packages can also be utilized alongside one another, similar to how MAP-RSeq can be utilized with EdgeR to obtain more information from the transcriptome data. Analysis software may vary in effectiveness based on the cell type. For example, Rajkumar et al. showed that EdgeR was superior compared to Cuffdiff2, DESeq2, and the two-stage Poisson model software analysis methods in determining DGE in mouse brain samples [47]. Software analysis tools can compensate for the limitations of current RNAseq methods [13]. The analysis software allows for normalizing the transcriptomic data, correcting errors associated with factors that are not biological, and smoothing the data by predicting missing values [41,48]. Some data analysis tools will also visualize the data at the cellular level through either cluster or trajectory analysis charts or at the genetic level through differential expression, gene set, or gene regulatory network analysis [48]. Regardless of the analysis method used, it is expected that the transcriptomic analysis of the RNAseq data can provide information that helps to uniquely identify different cell types. 

## 4. RNAseq Data Analytic Methods and Their Utilities

**Figure 2:**
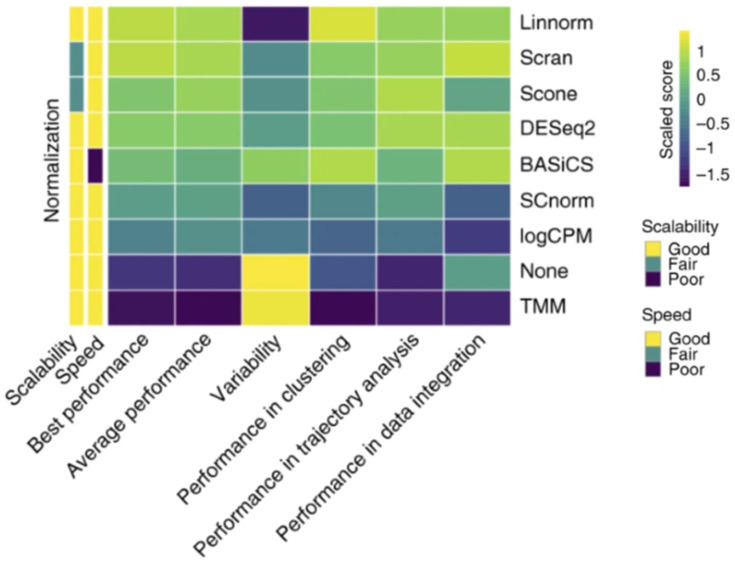
Normalization capabilities of several different RNAseq methods. Each method was scaled in comparison to the other tested methods, creating a scaled score for each method. Reproduced with permission from Tian, Nature Methods; published by Springer Nature, 2019 [51].

There are different methods to observe DGE and perform normalization depending on the RNAseq analysis method used. Bulk RNAseq normalization methods include TMM, DESeq2, and count-per-million, and bulk RNAseq differential expression methods include DESeq and EdgeR [49,51]. Linnorm, SCnorm, BASiCS, and scran are scRNAseq normalization methods, and MAST, Monocle, SCDE, and D^3^E are differential expression analysis methods mainly used for scRNAseq [50,51]. Linnorm and scran are the normalization methods that most consistently produced beneficial normalization results [51]. A comparison between normalization methods is shown in Figure 2. The differential expression analysis of scRNAseq data can utilize bulk analysis methods in some scenarios but requires a new characterization of the difference in expression to be established beyond having the average expression be a non-zero difference [49]. While normalization and differential expression are more effective when utilizing different methods for bulk and scRNAseq, clustering analysis software can be used on datasets from both sequencing methods. Clustering analysis methods include Seurat, RaceID3, RCA, SC3, and clusterExperiment [51]. Trajectory analysis methods such as DPT, Monocle2, Slingshot, SLICER, and TSCAN can be utilized for both bulk and scRNAseq as well but require distinct pseudotime paths from one cell type to another to generate each cell’s trajectory and overall lineage commitment [51]. While there are a few popular RNAseq analysis methods, such as EdgeR and DESeq2, for DGE, there is not a universal method. This is due to assumptions that must be made when using each analysis tool. Both EdgeR and DESeq2 assume that differentially expressed genes (DEGs) are rare and that the variance and signal intensity are independent of one another [54]. The transformation of the data must also occur in both methods when combining normalization and differential expression testing to minimize inconsistencies in results [54]. These assumptions may introduce errors in the data analysis. Therefore, each analysis method requires testing to find the optimal experimental conditions to minimize the error observed due to the assumptions made. The removal of outlier data points is one major source of error for some analysis methods, such as DESeq and DESeq2, due to the data manipulation of these methods [55]. DESeq utilizes a dispersion-mean trend to remove outlier data points, whereas DESeq2 uses Cook’s distance metric. In both instances, the removal of data points that fall outside of the distribution range occurs, which limits the discovery of new differentially expressed genes but still prevents false discovery [55]. Down-weighting the outlier data points rather than removing them from the dataset has the potential to reduce false discovery rates while still allowing for new DEGs to be discovered. Reducing false discovery errors not only improves the accuracy of the data but also allows for better comparison of the data between samples [55]. User error is another limitation of RNAseq analysis software. While DESeq2 and EdgeR are commonly used due to the strong results obtained, as well as the relative ease of use, novice users of the software packages could miss steps, such as checking the hypothesis of the methods or controlling data quality [54]. Tools such as SARTools have been created to streamline the use of DESeq2 or EdgeR to prevent user error [54]. This tool is used to ensure that all steps necessary for the proper use of the software are followed to prevent errors. Reducing error is beneficial to the software’s accuracy in producing normalization, cluster and trajectory analyses, and DGE results from RNAseq datasets.

RNAseq data analysis facilitates the identification of cell identity and the discovery of new cell subtypes by observing the DEGs and comparing them to previous results [56]. For instance, Clark et al. utilized the Smart-Seq2 data analysis method and 10x Genomics Chromium 3′ v2 platform software to characterize retinal cell types through the DGE analysis of scRNAseq transcriptomic data [57]. They observed gene expression changes throughout retinal neurogenesis while specifying different retinal cell types throughout development [57]. *CCND1*, *CDK4*, and *PAX6* were the gene markers used to specify retinal progenitor cells, while further specified cells, such as retinal ganglion cells and amacrine cells, were identified with *POU4F2*/*ISL1* and *TFAP2B* expression, respectively [57]. In Villani and co-workers’ work on characterizing blood cell types [58], the dendritic cells in their scRNAseq study were sequenced by utilizing Smart-Seq2 and analyzed with Seurat software. Two new subtypes of dendritic cells were discovered through sequencing analysis, finding the unique expression of *CD1C/BDCA-1^+^* cDC2 cells [58]. The first subpopulation expressed *CD1CA*, while the other expressed *CD1CB*, and neither subtype was related to any previously characterized dendritic cell type [58].

Machine learning uses algorithms and statistical computational models to analyze megadata. It has recently been utilized to observe the number of unique transcripts necessary to identify the cell type of a tested cell [53]. Aevermann et al. utilized NS-Forest v2.0 machine learning software to characterize cell type in a sNuc-Seq study of cells from each cortical layer of the middle temporal gyrus [53]. Machine learning utilizes known marker genes to classify cell clusters into specific cell types and further uses the genome expression information of these classified cell types to identify cell types in future datasets. In another study, Li et al. compared machine learning techniques for the cluster analysis of pancreatic cells from scRNAseq datasets [52]. The DESC deep neural network machine learning software was able to perform clustering stability and accuracy analyses comparable to other standard analysis methods, such as scVI, MNN, Seurat3.0, CCA, BERMUDA, and scanorama [52]. Furthermore, DESC was able to preserve the biological variation, remove the batch effect from the datasets, and allow for the biological interpretation of pseudo-temporal and discrete cellular structures [52]. Notably, cluster analysis and batch effect removal are usually accomplished with the simultaneous use of multiple different analysis software methods, while DESC can accomplish it with a single method [52]. Machine learning can present baseline information as to the number of unique genes displayed by a cell to be accurately characterized [59]. Peng et al. found that unique gene expression can be the result of RNA editing and not of unique cell types [59]. This creates the same issue as when the sequencing analysis does not properly filter outliers in that there will be false positives in determining DGE for that particular cell type [59]. Hence, predicted gene expression using machine learning–based methods after RNAseq should be validated by means of other biological characterizations.

## 5. Transcriptomics-Enabled Human Stem Cell Differentiation

RNAseq technologies have been extensively applied to the generation of a variety of cells and tissues from hPSCs in the past decade since differential expression characterization is vital for determining how cells develop and mature during the course of the stepwise differentiation of stem cells. The best way to analyze stem cell differentiation through RNAseq is to perform RNAseq at different differentiation stages to acquire important information regarding the specific genes crucial for the differentiation process [26,60,61]. Nair et al. used a 20-day stepwise differentiation protocol to differentiate hESCs into endocrine β-like cells and purified insulin-GFP-expressing β-like cells by flow cytometry, followed by the aggregation of these immature insulin-GFP+ cells with extended culture. They compared the transcriptome differences between the hESC-derived immature β-cells after 20 days without aggregation, cells after aggregation and extended culture, and native β-cells isolated from adult human islets using RNAseq analysis. They found that the isolation of hESC-derived immature insulin-GFP-expressing cells and the aggregation of these cells into islet sizes with extended culture allowed the generation of mature β-cells with functions similar to those of human islet β cells [62]. These stem-cell-derived cells showed dynamic insulin secretion and increased calcium signaling in response to insulin secretagogues. Wang et al. utilized EdgeR to determine DEGs in human mesenchymal stem cell (MSC) differentiation into endothelial cells. Differentially expressed genes in endothelial cells compared to MSCs include *HIPK2*, *GREM1*, *LEF1*, and *EFNB2* [63]. The upregulations of these genes, along with seven other genes, namely, *ADGRA2*, *CHRNA7*, *LRG1*, *NTRK1*, *S100A9*, *MMRN2*, and *RAPGEF3*, in MSC differentiation toward endothelial cells is an indication of endothelial specification by RNAseq analysis [63]. Through RNAseq analysis at different stages of MSC differentiation, specifically toward endothelial cells, the study found that ECM organization, angiogenesis, blood vessel morphogenesis, and growth-factor- and ECM-binding pathways all had enriched expression at the later stage of differentiation [63].

**Figure 3:**
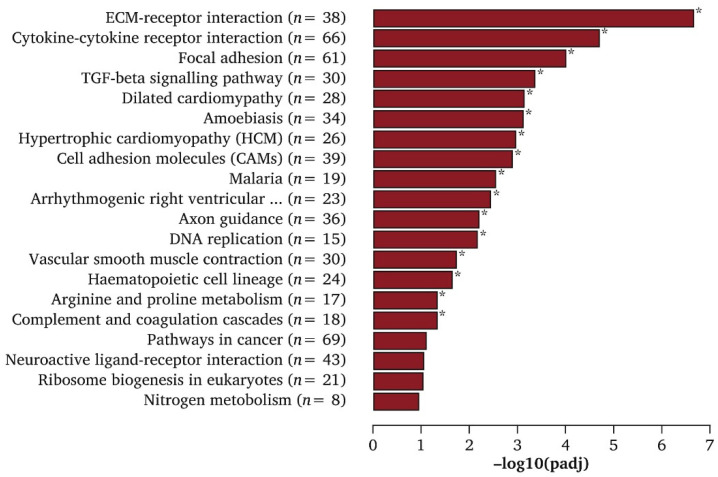
Top KEGG enrichment pathways of differentially expressed genes (DEGs) in endothelial cells generated from MSCs. * Indicated significantly enriched KEGG pathway terms found in induced endothelial cells compared with MSCs. Reproduced with permission from Wang, European Journal of Vascular and Endovascular Surgery; published by Elsevier, 2020 [63].

The process of analyzing RNAseq results at varying time points is known as temporal RNAseq. DyNB and DESeq analysis methods were utilized by Äijö et al. to assess temporal RNAseq data [60]. Both analysis methods are run with an assumption that ignores correlations between time points, which allows for the testing of each time point for DEGs independently of time. This reduces the need for the regularization of the data and provides estimations of gene expression throughout differentiation, as well as shows transcriptome responses to differentiation [60]. Th17 cells are a subclass of T helper lymphocyte cells that express interleukin 17 (IL17) [60]. *ISG20* and *IL17A* are known to have roles in Th17 cell function. Their expression was initiated after 48 h post–TH17 differentiation and showed increased expression thereafter, as quantified by temporal RNAseq analysis [60]. By analyzing stem cells at different time points, one can determine how gene expression changes between each time point. This can show not only the differentiation pathway of the cell throughout its lineage commitment but also reveal new cell subpopulations or intermediates during differentiation. Wang et al. demonstrated that signaling pathways involved in ECM–receptor interactions, TGF-β, and cytokine–cytokine-receptor interactions had the highest expression for MSC differentiation toward endothelial cells [63]. The top Kyoto Encyclopedia of Genes and Genomes (KEGG) signaling pathways for MSC-to-endothelial-cell differentiation are shown in Figure 3. The TGF-β pathway is consistently downregulated across associated genes, such as *NOG*, *ID4*, *INHBA*, *INHBB*, *INHBE*, *THBS2*, *BMP8B*, *COMP*, and *CHRD*, during endothelial specification [63]. However, ECM organization genes such as *MMP1*, *ELANE*, *CTSG*, *MMP10*, *KDR*, *MYF5*, *VWF*, *PDGFB*, and *COL17A1* are all significantly upregulated for MSC-to-endothelial-cell differentiation [63]. These results indicate that ECM interaction and organization-related gene upregulation and TGF-β inhibition are important for endothelial specification. RNAseq can also increase the knowledge of the differentiation mechanisms involved in the specification of cells derived from stem cells [63]. Such sequencing could give insight into the regulatory networks that are utilized to promote cell specification and lineage commitment [26]. This can be useful in determining the balance between the proliferation and differentiation of stem cells [26]. Certain data analysis techniques are also able to discern cell cycle processes that are closely aligned to cellular differentiation [61]. Temporal RNAseq allows for more complete data acquisition compared to normal sequencing analysis. However, cell differentiation still presents issues even with temporal sequencing analysis. For instance, single-cell temporal RNAseq can reveal the differentiation of a single cell, but there is no guarantee that analyzing at the same time points during the differentiation of another cell of the same type will yield the same results. The second cell could differentiate at a much different rate, suggesting that comparing time points is not completely accurate. As an alternative approach, same-time temporal modeling eliminates this issue, as there is no need for replicates to observe each different differentiation step [60]. Same-time temporal sequencing is the process of sequencing cells simultaneously, each at a different stage of differentiation into the desired cell type. This removes variable differentiation rate errors since specific cells are selected at specific stages of differentiation for sequencing and data analysis [60].

Table 3 exhibits diverse RNAseq methods that aid in hPSC differentiation into varied cell types. Collin et al. focused on the development of retinal organoids from hESCs and further characterized the cell populations within the organoid through temporal scRNAseq using integrated fluidic circuits [64]. Transcriptomic data for multiple time points during differentiation were collected to characterize the orderly appearance of retinal cell types as organoid development progressed [64]. Additionally, scRNAseq has been applied to hESC differentiation into pancreatic β-cells [65] and heart organoid tissue [66] (Table 3). hESC-to-β-cell differentiation was tracked at multiple points throughout differentiation to characterize genomic expression changes during β-cell development [65]. Spatiotemporal scRNAseq was utilized to map the cell type distribution and genome expression in the human heart during embryonic development to better understand cardiac morphogenesis [66]. Spatiotemporal scRNAseq has also been applied to understanding how hypoxia plays a role in iPSC differentiation toward liver bud cells throughout organoid development [67]. Kidwai et al. assessed the progress of hESC and iPSC differentiation into osteogenic progenitor cells through bulk RNAseq and focused on transcriptomic differences between osteogenic progenitors derived from the paraxial mesoderm, lateral plate mesoderm, and neural crest [68]. Tsujimoto et al. applied both bulk and scRNAseq to characterize iPSC differentiation toward kidney-lineage-specific cells, such as metanephric and mesonephric nephron progenitors, as well as ureteric bud cells, to gain a better understanding of the mechanisms that are behind kidney development [69]. They focused on early renal development to characterize the origin of embryonic kidney cell types derived from the intermediate mesoderm to better characterize early kidney development in kidney organoids. Furthermore, RNAseq and transcriptome characterization can be utilized for disease treatment. Wang et al. assessed the transcriptome data of endothelial cell differentiation from MSCs by utilizing bulk RNAseq, aiming to understand the mechanism underlying differentiation for potential cell-based treatment that can reduce ischemic symptoms in patients with peripheral artery disease [63] (Table 3).

Huang et al. focused on analyzing the transcriptomes of pancreatic β-cells derived from iPSCs, looking specifically for DEGs [70]. iPSC generation of β-cells was shown to be possible in both healthy patients and diabetic patients. The main process of iPSC-to-β-cell differentiation involved the generation of embryoid bodies from the initial iPSCs, the differentiation of embryoid bodies into multipotent progenitors, and the induction of β-cells from the multipotent progenitors. Differentiation was aided by increased signaling via the TGF-β, PI3K-AKT, and MAPK signaling pathways in later pancreatic differentiation toward a β-cell fate [70]. Specific gene expression favoring β-cell differentiation includes genes associated with each of the highly expressed signaling pathways. The β-cell fate is tied to the high expression of signaling pathway genes, such as TGF-β genes (*TNF*, *TGFB2*, and *THSB1*), PI3K genes (*ITGB4*, *FGF3*, *LAMA1*, *JAK2*, and *FLT1*), and MAPK genes (*TNF*, *FGF3*, *TGFB2*, and *FGFR2*) [70]. Liang et al. also focused on how transcriptomic expression can benefit stem cell differentiation but focused on dopaminergic progenitor generation from hESCs [71]. Dopaminergic progenitors are located in the midbrain and are part of the differentiation pathway that progresses from hESCs to radial glial cells to early floor plate progenitor cells to dopaminergic progenitor cells. Favorable transcriptomic signatures have been discovered for each step in the differentiation process from hESCs to dopaminergic progenitors. The initial high expression of *SOX2*, *FABP7*, *FEZF1*, *TCF7L2*, and *HMGB2* promotes radial glial specification and eventually gives way to the high expression of floor plate markers such as *LMX1A*, *CORIN*, *OTX2*, and *FOXA2* [71]. The final high expression of dopaminergic progenitor markers, including *TH*, *NR4A2*, and *PITX3*, demonstrates effective differentiation into dopaminergic progenitor cells from hESCs [71].

Aside from characterizing known cell types and discovering new unique cell subtypes, RNAseq analysis has been used to observe how substrates alter cellular gene expression and in cell differentiation studies [26,72]. Carrow et al. reported how the presence of synthetic clay nanosilicates can alter the cellular transcription profile in human MSCs and prompt differentiation toward an osteochondral cell fate [72]. Gene expression analysis indicates that nanosilicate introduction negatively impacts the expression of the multipotent and motility markers *WNT5A*, *AFAP1*, *SOCS5*, and *INHBA* in MSCs while increasing the expression of the bone and cartilage development genes *COMP*, *COL1A1*, *ACAN*, and *COL11A* [72]. The data support the theory that nanosilicates alter the transcriptome expression in human MSCs toward an osteochondral cell fate. Lange et al. developed a toolkit, named CellRank, for single-cell fate mapping after scRNAseq. The toolkit helps to predict the cell fate trajectory during cellular differentiation [73]. It predicted both the terminal state and fate potential of the analyzed cell. This is accomplished by looking at the genome expression in the analyzed cell and comparing it to all of the possible cell types that the progenitor could terminally differentiate into [73]. Based on DGE, the analysis software predicts the most likely cell fate. While this is not an exact prediction, the software is capable of determining the level of uncertainty in the lineage commitment prediction [73].

## 6. Transcriptomics-Aided Discovery of Intrinsic Factors Influencing hPSC Lineage Commitment

Transcription factors bind to DNA and directly influence target genes’ transcription and expression in the cells [74]. Hence, transcription factors play a pivotal role in regulating cellular differentiation. Cells cannot further specify the desired lineage without expressing specific transcription factors [75]. Reimold reported that the transcription factor XBP-1 is selectively and specifically required for the generation of plasma cells. Heller et al. analyzed RNAseq and chromatin immunoprecipitation-sequencing (ChIPseq) data to understand transcriptional and regulatory mechanisms during hPSC pancreatic development [76]. They discovered that *GATA6* and *FOXA2* are responsible for inducing hESC differentiation toward a definitive endoderm cell fate, which will later differentiate into pancreatic and endocrine progenitors alongside the expression of other transcription factors, including *ONECUT1*, *PDX1*, *SOX9*, and *NKX6.1* [76]. Using the RNAseq approach, Jennings et al. identified transcription factors responsible for initiating the development of either the pancreas or liver [77]. *PDX1*, *GATA6*, *FOXA2*, *HNF1B*, and *ONECUT1* control pancreatic endoderm commitment from hPSCs, while *HNF4A* and *FOXA* are key transcription factors regulating the onset of liver specification from hPSCs [77].

Transcription factor enhancement or repression could make a difference in late-stage differentiation into two separate committed cell lines. Yagi et al. demonstrated how *GATA3* expression influences the late-stage differentiation of CD4+ T cells into either Th1 or Th2 cells [78]. As transcription factors are capable of binding to DNA to directly influence genes that are transcribed and expressed in a cell, gene expression is directly influenced and controlled by transcription factors. Ge et al. confirmed that *ATOH7*, *OTX2*, *POU4F2*, and *ISL1* all influence the differentiation of retinal progenitors toward a retinal ganglion cell fate by attaching to the genome and influencing gene expression through RNAseq data analysis [79]. Due to the significant relationship between transcription factor presence and specific gene expression, transcription factors are vital for cell lineage commitment.

**Figure 4:**
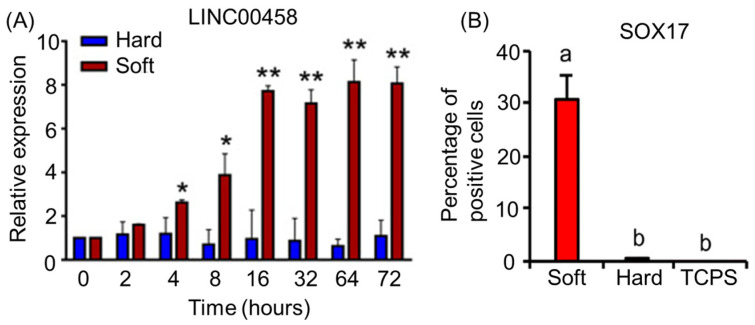
(**A**) Expression of lncRNA *LINC00458* in hPSCs cultured on hard (165 kPa) or soft (3 kPa) substrates. Cells were cultured on soft and hard substrates for 3 days. RNA was isolated at the indicated time points, and *LINC00458* expression was detected by qRT-PCR. The results are presented as means ± SD of triplicates. ** *p* < 0.005, * *p* < 0.05, two-sided Student’s *t* test. (**B**) Percentage of SOX17+ cells in hPSCs grown on substrates with varied stiffnesses. The results are presented as means ± SD of triplicates. One-way ANOVA (*n* = 3 independent experiments). Different letters indicate significant differences, and the same letters indicate no significant differences. The data are adapted from Chen et al. [84].

Notably, RNAseq technologies allow the sequencing of not only transcription factors but also long non-coding RNA (lncRNA) and microRNA (miRNA). lncRNA and miRNA have been implicated in cellular differentiation and are able to regulate gene expression [80,81,82,83]. miRNAs are small RNA transcripts, and lncRNAs make up the majority of the transcriptome [82,83]. However, lncRNAs have a complex overlap of transcripts that makes the discovery of their function difficult [82]. Through RNAseq analysis, Chen et al. discovered that the expression of *LINC00458*, a lncRNA, induces hPSCs toward an endodermal cell fate, provided that the cells are cultured on soft substrates [84]. The influence of substrate stiffness on *LINC00458* expression in cultured hPSCs is shown in Figure 4A. Different expression levels of this lncRNA resulted in varied endodermal cell commitment, as quantified by SOX17 protein expression levels (Figure 4B) [84]. Increasing the stiffness of the substrate has been shown to negatively influence *LINC00458* expression, stunting differentiation into endodermal lineages. Soft substrates are favorable for inducing *LINC00458* expression, which in turn interacts with *SMAD2/3* within the nucleus. The knockout of *LINC00458* also resulted in the loss of endodermal marker genes such as *FOXA2* and *SOX17*, indicating the importance of this lncRNA in hPSC-to-endoderm differentiation [84]. Jha et al. showed the lncRNA *GATA6-AS1* is necessary for proper differentiation from hPSCs to cardiomyocytes. This lncRNA expression is directly related to the expression level of the *GATA6* gene [80]. It was shown that the knockout of *GATA6-AS1* restricted cardiomyocyte lineage specification through the direct loss of *GATA6* expression, which is essential for cardiomyocyte development from hPSCs [80]. On the other hand, *miR-17~92* has been shown to repress *CXCR5* expression in developing T cells, which results in inhibited T-cell generation and migration. However, *miR-17~92* is also necessary for the repression of *TH17*, *Th22*, and other associated incorrect lineages’ gene expression during T-cell differentiation into T follicular helper cells [83]. These repressed genes include *RORA*, *CCR6*, *ILLR2*, *ILLR1*, and *IL22* [83]. By means of miRNAseq and RNAseq, miRNA has also been discovered to impact human placental development studies using the early stages of placental samples [81]. These studies strongly suggest that RNAseq techniques facilitate the discovery of miRNAs that play a role in the differentiation and development of cells.

## 7. Transcriptomics Applied to Disease Physiology Studies Using Patients’ iPSC-Derived Cells for Regenerative Medicine

**Figure 5:**
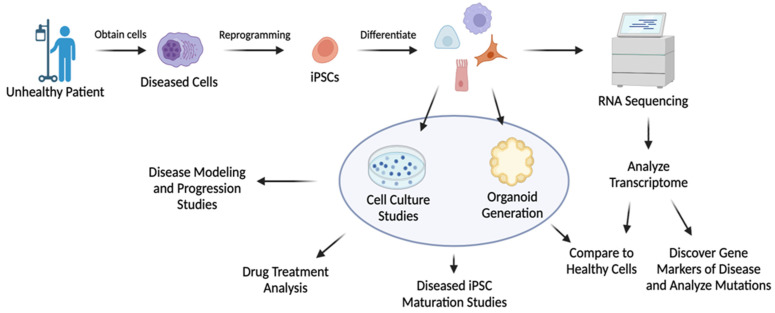
Model of diseased iPSC generation and its use for regenerative studies and disease characterization. Created using Biorender.com.

Transcriptome analysis is beneficial for understanding disease and providing insight into potential regenerative medicines capable of mitigating or treating disease. This commonly encompasses utilizing iPSCs derived from patients’ tissues to generate diseased organoids for research (Table 4) [85,86,87,88,89,90,91]. Figure 5 summarizes the key steps in disease modeling through a combination of iPSC technology and transcriptomics for regenerative medicine. For instance, iPSC-derived brain cells have been utilized to mimic the progression of Alzheimer’s disease (AD) to study the transcriptomic effect on cells. These patient-derived brain cells obtained from iPSC differentiation showed genotypes and phenotypes associated with AD [85]. Zhao et al. generated cerebral organoids from AD dementia patient-derived iPSCs. Transcriptomic profiling revealed that the cerebral organoids originating from AD patients showed abnormal cytoplasmic RNA granules and deranged RNA metabolism [92]. The studies provide a more in-depth understanding of the disease.

Another disease in which transcriptomic analysis has been utilized for potential regenerative medicine is Parkinson’s disease (PD). PD studies currently lack dopaminergic neuron samples, as these neurons die off at an increasing rate with the progression of the disease, leaving few neuronal samples post-mortem [86]. iPSCs derived from fibroblasts have been generated with the *PINK1* gene mutation *ILE368ASN* to imitate PD dopaminergic neurons [86]. These mutated neurons were utilized to analyze the dysregulation of neuronal function as a result of PD (Table 4). Novak et al. theorized that PD is not the result of a single mutation but rather the effect of transcriptomic dysregulation from several interconnected genes and signaling pathways [86]. Azevedo et al. also analyzed PD through iPSCs but instead analyzed how myelination was prevented during oligodendrocyte specification and maturation [93]. This loss of maturation in PD patients leads to a lower transcriptional contribution of oligodendrocytes compared to healthy controls [87]. The utilization of iPSCs developed from PD patients led to the discovery that alterations in the cell’s transcriptome are associated with the loss of myelination in oligodendrocytes [93]. For example, mutations in the *SNCA* gene are known to play a role in the loss of oligodendrocyte myelination in PD [93]. *A53T α-synuclein* and *G2019S LRRK2* mutations have also been implicated in the formation of PD [87]. *LMX1B* and *OTX2* are suggested to influence PD pathogenesis [87]. Numerous genes were found to be enriched or depleted in PD-patient-derived oligodendrocytes through transcriptomic analysis, suggesting the involvement of these genes in PD. iPSC-derived oligodendrocytes also showed the involvement of the inflammatory components C4b and HLA proteins, promoting oligodendrocyte immune reactivity rather than myelination [93]. Moreover, studies in iPSC-derived PD models are able to determine familial-PD- and sporadic-PD-related gene expression, including the expression levels of miRNA and piRNA, for understanding PD gene deregulation. miRNA and piRNA transcriptome expression is altered as a result of PD presence (Table 4) [87]. It was shown that 26, 34, and 40 different miRNAs were dysregulated in the PD group compared to the healthy control group for fibroblasts, iPSCs, and neurons, respectively. The increased expression of *LET7*-family miRNAs occurs consistently in PD patient samples. *SINE*- and *LINE*-family piRNA overexpression is associated with PD, as diseased neurons cannot silence these genes properly [87]. Temporal RNAseq analysis has been utilized to analyze DEGs for mutated PD dopaminergic neurons [86]. Dopamine metabolism genes *TH* and *DCC* were significantly expressed in mutated dopaminergic neurons alongside *NES* and *VIM*, which are cytoskeletal protein genes. Both *NES* and *VIM* are associated with cytoskeletal transport, a process commonly impacted by PD [86]. Other common PD-related genes include *LGI1* and *CNTNAP2*, which are both dysregulated throughout disease progression, although they do not have direct links to the disease [86].

Recently, transcriptome analysis became a powerful tool for regenerative medicine applications in diabetes research thanks to the continual improvement of protocols for hPSC–endocrine cell differentiation [2,3,4,94,95,96,97]. Technologies for iPSC differentiation into insulin-secreting β-cells have been applied to generate β-cells or endocrine tissue from patients suffering from different subtypes of diabetes, such as type I diabetes (T1D), type II diabetes (T2D), and monogenic diabetes (MD) (Table 4) [88,98,99,100]. Memon et al. focused on utilizing T2D-derived iPSCs to study the mechanism behind insulin resistance in T2D [89]. RNAseq analysis of insulin-resistant iPSCs discovered the downregulation of *L1TD1*, *RIF1*, *MYSM1*, *ZNF195*, *ZNF208*, and *ZNF770* compared to healthy controls [89]. Upregulated genes in the insulin-resistant group compared to healthy controls include *AGRN*, *SLFN13*, *CD74*, *SEMA6B*, *SLC22A17*, and *TMEM151B* [89]. The T2D iPSC group had increased lactate secretion and increased oxidative stress in the cells, suggesting lowered oxygen availability to the diseased islet cells. Oxidative stress also induces mitochondrial dysfunction, which compounds the impact of insulin resistance on affected islet cells [89]. A combination of diseased iPSC-derived cells and transcriptome analysis has been utilized to understand the mechanism and transcriptomic changes causing diabetes. For example, it helped to reveal the genetic mechanism underlying transcription factor *PDX1*-mutation-associated impaired glucose tolerance in patients with an increased risk for diabetes [98]. Using a *PDX1*-mutant-derived iPSC line and subsequent differentiation, the study deduced that amino acid mutations in *PDX1* impair pancreatic endocrine formation and β-cell function, contributing to a predisposition for diabetes [98]. The downregulation of the *PDX1*-bound genes *MNX1*, *MEG3*, and *CES1* is attributed to the lowered β-cell differentiation ability [98]. Augsornworawat et al. discovered that β-cell maturation occurs in both iPSC- and hESC-derived islets post-implantation [101]. Maturation was assessed based on the comparison of β-cell maturation gene expression levels before and after implantation through RNAseq data analysis. The β-cell maturation genes *INS*, *MAFA*, *MNX1*, *SIX2*, and *G6PC2* were all expressed in higher quantities in the post-transplantation group. The increased maturation and functionality of the transplanted islets suggest that native tissue gene expression can be acquired once the iPSC-derived cells are transplanted in vivo. Both α- and β-cells undergo increased maturation once they are transplanted in vivo, further suggesting that the in vivo environment and native tissue influence genome expression toward more mature cells [101]. Furthermore, RNAseq data analysis allowed the identification of transcriptomic abnormalities. Mutated transcription factors can be corrected through genome editing using tools such as CRISPR/Cas9 to restore the proper functionality in the diseased islets [91,102]. These genes include but are not limited to *HNF1A* [103], *INS* [99,104], and *GATA6* [105].

In addition, hypertrophic cardiomyopathy (HCM) is another disease in which transcriptomic information can be uncovered from diseased iPSC-derived cells. Patient tissue was utilized to generate iPSCs, which were then developed into cardiomyocytes for transcriptomic analysis studies focused on discovering genes related to HCM functionality (Table 4) [90]. Approximately 50% of HCM patients carry mutations in sarcomere genes, such as *MYH7* and *MYBPC3* [106]. The *MYBPC3 c.1928-569G* > *T* mutation was discovered as one of the causes of HCM through iPSC-derived cardiomyocyte transcriptome analysis. This mutation results in aberrant splicing, which is commonly undetected in genetic testing [90]. Not only does this method uncover a new cause for HCM, but it also opens the door for research into the therapeutic inhibition of aberrant splicing, which could treat the disease [90]. iPSC-derived cardiomyocytes have also been used for disease modeling and RNAseq analysis of congenital heart disease (CHD). Xu et al. utilized iPSC-derived cardiomyocytes to model hypoplastic left heart syndrome by analyzing the impact of the defects caused by the disease [107]. scRNAseq showed a correlation between the number of DEGs and disease severity compared to the healthy control group. An increased number of DEGs indicated the increased severity of disease progression [107]. The iPSC-derived cardiomyocytes that were from patients with less severe disease progression had gene expression more similar to the healthy control iPSC-derived cardiomyocytes when compared to patients with more severe disease progression. Many of the DEGs correspond to signaling pathways in mitochondria, hypoxia, cell death, or apoptosis. These pathways all corresponded to increased mitochondrial dysfunction in the diseased groups that caused severe oxidative stress and increased cell death [107].

**Table 4:** Patients’ iPSC-derived cells for disease modeling by means of transcriptomics.

Disease Type	Patient Cells Utilized	Final Cell Types	Purpose	References
Alzheimer’s disease	iPSCs	Neural progenitors, astrocytes, microglia, and oligodendrocytes	Brain cells generated from AD patients’ iPSCs to mimic disease progression by transcriptomic analysis	[85]
Parkinson’s disease	iPSCs	Dopaminergic neurons	A PD patient’s dopaminergic neurons generated from iPSCs for scRNAseq analysis	[86]
	iPSCs	Oligodendrocytes	Transcriptome analysis of PD patient iPSC-derived oligodendrocytes to determine cause of reduced myelination	[93]
	iPSCs	Midbrain neurons	iPSC-derived model to study the effect of mRNA, miRNA, and piRNA on PD progression	[87]
Diabetes	iPSCs	Insulin-resistant iPSCs	T2D-patient-derived iPSCs to study insulin resistance mechanisms such as increased oxidative stress and lactate secretion	[89]
	iPSCs	Pancreatic β cells	Patient iPSCs to study the effect of mutant PDX1 expression on impaired glucose tolerance	[98]
	hESCs and iPSCs	Pancreatic α, β, and δ cells	iPSC-derived islets further mature after implantation into the body based on RNAseq data analysis	[101]
Hypertrophic cardiomyopathy	iPSCs	Cardiomyocytes	Transcriptomic analysis of iPSC-derived cardiomyocytes from diseased patients	[90]
Congenital heart disease	iPSCs	Cardiomyocytes	Modeling hypoplastic left heart syndrome through iPSC-derived cardiomyocytes	[107]

## 8. Challenges and Limitations

A limitation of current RNAseq is the necessity of cDNA reverse transcription for the sufficient scale-up of genomic information [13]. The challenge with utilizing cDNA comes with many manipulation steps necessary for RNAseq. Manipulation is necessary to remove second-strand cDNA from strand-specific sequencing, account for cDNA library generation-related dissociation, and synthesize cDNA without independent primer synthesis. Some genes are uncooperative with RNAseq and will not provide accurate results. Uncooperative genes are typically small in base-pair number and produce a significant batch effect when sequenced utilizing different library preparation techniques [108]. When using unique versus multiple alignment, some genes seemed to be differentially expressed in one of the methods rather than both [108]. This occurrence suggests that the differentially expressed gene could be a false-positive value, which throws off the accuracy of the data interpretation. On top of uncooperative genes, some RNAseq methods have limited reproducibility [109]. NOIseq is one analysis method that cannot be reproduced, as the noise from one dataset to another is not consistent [44]. Another major challenge to RNAseq analysis is analyzing low-input samples or samples that have become degraded [34]. The majority of analysis methods are limited to analyzing full transcripts, and incomplete transcripts lead to a highly inaccurate interpretation of the transcriptome. For more accurate results, studies must be completed using productive RNAseq and analysis methods, together with experimental confirmation. For degraded samples, more effective analysis methods include RNase H and Ribo-Zero [34]. Due to the availability of different types of analysis software, only a limited number of methods have been significantly investigated in a research context [109]. More studies will have to be completed to validate the accuracy of the software programs and the resulting transcriptomic data. 

Another major limitation of RNAseq and the associated data analysis is the variability in the sequencing results [108,110]. RNAseq has had a limited number of sample replicates for many cell types, resulting in the lack of sufficient data to compare the new samples to [55]. Efforts must be made to increase the number of samples sequenced for each known cell type to increase the accuracy of the analysis and comparison to previous methods. Increasing the sample size will decrease the deviation of gene expression for each cell type and allow for the comparison of new samples to a more uniform dataset of genome libraries. The false discovery of DEGs or new cell subtypes is another source of error [55,108]. This can partially be caused by RNA editing of a cell’s transcriptome [59]. No two cells are identical due to biological variations regarding how RNA is transcribed, which could lead to increased error for the analyzed dataset. Moreover, environmental changes of any magnitude can potentially alter the identification of DEGs during RNAseq analysis [111]. This could be from using different batches for sequencing or having the batches collected at different time points within the experiment. Environmental changes could also occur during RNA isolation from contamination or slight variations in how the RNA libraries are obtained and prepared [111]. Temporal RNAseq analysis is limited by the variability between replicates due to differentiation efficiency [60]. This can throw off the data observed during stem cell differentiation analysis. Some analysis software, such as DyNB, has implemented filtering that accounts for the variable timing of differentiation, which significantly reduces the error [60].

On the other hand, RNAseq analysis for genes that have low copy numbers or are expressed at low levels presents challenges. Filters must be applied to separate the low-copy-number genes from the outlier genes and other associated noise [112]. Noise is the result of biological variation within each cell as well as transcript loss during the preparation of the cDNA library [113]. Genes from regions of low density can sometimes be falsely classified as noise due to the assumptions necessary to run RNAseq analysis [113]. Hence, properly eliminating noise and distinguishing noise from a low copy number of genes are pivotal to obtaining highly accurate DGE and cluster formation analysis results. Furthermore, the sequencing depth and the number of genes detected in each biological sample influence RNAseq analysis accuracy [112,114,115,116]. The sequencing depth, otherwise known as the size of the sequenced library, varies from sample to sample due to biological variation between samples [114]. Therefore, normalization must be performed to accurately compare different genes and datasets. Zyprych-Walczak et al. investigated multiple data normalization methods, including the EdgeR packages TMM and Upper Quartile, the DESeq package Median, the EBSeq package Quantile, and the PoissonSeq package PoissonSeq normalization [114]. They noted that TMM performed poorly, but there was not one method that performed significantly better than the others out of the remaining methods [114]. It was also noted that bias and variance should be analyzed as a part of normalization in order to find a method that is best suited to analyze the dataset and prevent differential expression errors [114]. Furthermore, current stem cell differentiation methods cannot obtain homogeneity of the final derived cellular product. A portion of subpopulations of cells still differentiate into undesired cell types. By determining the genomic expression of the cells, the goal is to gain an understanding of how the cells differentiate and why subpopulations form. Once this is known, the undesired cell clusters can be eliminated to achieve the desired differentiation pathways.

## 9. Future Direction of RNAseq-Aided Stem Cell Differentiation

The future of RNAseq should look toward addressing the limitations of the current methods. Sample multiplexing for scRNAseq is one method that would enable the super-loading of single cells and the unbiased analysis and discovery of cell types [117,118]. Multiplexing scRNAseq has advanced to the point where over 10^5^ cells can be sequenced at once [117]. RNAseq has a variation in read depths when using different sequencing methods, which results in a different number of transcripts generated by sequencing. This variability is an issue for comparison and accuracy. This can increase the error in the analysis between that dataset and another that utilized a different analysis method. Normalization methods such as those described by Zyprych-Walczak et al. must be perfected to generate accurate and comparable RNAseq data [114]. There should be a focus on finding a universal method for RNAseq and analysis that provides accurate results regardless of experimental conditions [119]. This necessitates the generation of a method that has little to no assumptions necessary to properly characterize the dataset. Assumptions limit the accuracy of the data analysis and further restrict comparisons between datasets analyzed by different methods [54]. Finding a universal method will not only standardize the experimental procedure necessary for proper sequencing but also eliminate the variability and error in the results generated and compared by using different sequencing and analysis methods. 

Spatial single-cell transcriptomics is another aspect of RNAseq that can be focused on more in future stem cell research. It provides cell type information at the spatial level in histological sections. Analyzing the RNAseq data in this manner allows for heterogeneity and tissue architecture to be observed in a spatial context at the cellular level [28,29,30,31]. Moncada et al. were able to map multi-cell-type locations and identified that populations of ductal cells, macrophages, dendritic cells, and cancer cells are spatially restricted, along with distinctly co-localized with other cell types, using a combination of spatial transcriptomics and scRNAseq techniques [29]. The combination of these pseudo-spatial techniques, called multimodal intersection analysis, was used to detect cell type and subtype enrichment across different locations within a specific tissue microenvironment, such as pancreatic ductal adenocarcinoma [29]. Spatial transcriptomics has been applied to embryos to characterize and identify the location and makeup of the developing organs [28]. Liu et al. utilized the Seurat3.2 module SCTransform for data normalization and stabilization, while spatial variability and differential expression were determined by SpatialDE and ToppGene, respectively, for their “deterministic barcoding in tissue for spatial omics sequencing” [28]. The method was utilized to characterize the spatial clustering of major cell types, including the heart, telencephalon, neural tube, hindlimb bud, and branchial arches, in a developing embryo.

Spatial transcriptomics can also be paired with temporal transcriptomics in RNAseq analysis to give an even more complete picture of gene expression over both space and time [28,31]. This approach allows for the analysis of cell interactions that drive differentiation and the transcriptomic profile associated with those interactions at specific locations over the time period of development. Hence, the lineage commitment of specific parts of the organ, as well as each cell cluster’s progression into specific cell types, can be tracked and mapped [28]. The prospect of analyzing RNA expression and cellular interactions over space and time allows for a better understanding of cellular interactions and development. Spatiotemporal RNAseq technology has been applied to viewing organ development and differentiation in chicken models [28], mouse organogenesis [32], human cancer research [29], thymus organogenesis in human embryos [33], and human fetal digestive tract development [31]. However, its application to stem cell differentiation is currently limited due to the high complexity of differentiated cells and the cost of spatiotemporal RNAseq [28]. This technology should be further improved to analyze developing organs and differentiating cells. The next steps should be toward implementing organogenesis observation with RNAseq to further characterize development in a spatiotemporal manner.

Taken together, stem cell differentiation has yet to accomplish pure lineage commitment toward the desired mature cell types. Global transcriptome analysis by RNAseq provides a powerful characterization tool to ensure the systematic assessment of the differentiated cells’ identities and identify key signaling pathways critical for the improvement of lineage commitment. Spatial and temporal transcriptomics alongside RNAseq would give more complex data involving cell–cell interactions, tissue architecture, and the regulatory networks that control stem cell differentiation and maturation. RNAseq would assist in the full characterization of human organoids to benefit in vitro organoid development for potential implantation into the body in the near future.

## Figures and Tables

**Table 1:** RNAseq techniques applied in current research.

RNAseq Technique	Description	Advantages	Limitations	Methods	References
Bulk RNAseq	Measures genome expression of bulk sample containing many cells and averages expression	Lower data variability between samples, cost-effective	Cannot distinguish unique cell populations	SMARTer SuperScript II reverse transcriptase from Invitrogen Prime-seq method	[21,22,24,25]
scRNAseq	Measures genome expression of individual cells	Distinguishes unique cell populations	Cannot accurately distinguish gene expression from multinucleated cells	Tube-based sequencing methods: SMARTer Ultra Low RNA Kit, TransPlex kitMicrofluidic-based sequencing methods: SMARTer cDNA synthesis utilizing the C1 microfluidic system	[21,22]
sNuc-Seq	Measures genome expression of individual nuclei	Best method for analyzing multinucleated cells	Does not consider genomic information from outside of the nucleus	Tube-based sequencing methods: SMARTer Ultra Low RNA Kit, TransPlex kit Microfluidic-based sequencing methods: SMARTer cDNA synthesis utilizing the C1 microfluidic system	[21,22]
Temporal RNAseq	scRNAseq method for analyzing genome expression of individual cells at different time points	More complete data acquisition compared to scRNAseq, tracks gene expression of differentiating and maturing cells	Very expensive; variable differentiation rate can lead to transcriptome inconsistencies between cells by assuming they are at the same differentiation step	Next-maSigPro Modeling the Poisson distribution’s rate parameter	[26,27]
Spatial RNAseq	scRNAseq method for analyzing genome expression of individual cells within a measured cluster to find location of specific gene expression	Provides cell transcriptomic information at the spatial level within histological sections, measures heterogeneity of genomic information and tissue architecture at a cellular level	Very limited current applications in research, no applications in stem cell differentiation	DBiT-seq for spatial omics sequencing using formaldehyde-fixed tissues Spatial sequencing performed with 2100 Bioanalyzer from Agilent with Qubit dsDNAHS Assay Kit from Life Technologies and sequenced with Illumina NextSeq sequencer	[28,29,30,31]
Spatiotemporal RNAseq	scRNAseq method for analyzing genome expression that combines spatial and temporal RNAseq	Combines advantages of spatial and temporal sequencing to obtain genomic information and tissue architecture of a histological section over time	Very expensive, very limited current applications in research, no applications in stem cell differentiation	10x RNAseq Visium spatial transcriptomic platform utilizing PHATE trajectory analysis	[28,29,31,32,33]

**Table 2:** RNAseq analysis software and associated software packages.

Software	Analysis Packages	Description and Common Uses	References
R	vegan	Determines saturation of a sample	[42]
R	EdgeR v3.2.4, limma, DESeq, DESeq2 v.1.0.19, baySeq, voom, Myrna, EBSeq, two-stage Poisson model, MAST v.1.0.5, monocle, SCDE	DGE analysis of datasets	[43,44,45,47,49,50]
Python v.2.7.6	D^3^E	DGE analysis of datasets	[49,50]
C++	Cuffdiff2 v.2.1.1	DGE analysis of datasets	[47]
R, Python v.2.7, Java v.1.6.0_17, PERL v.5.10.0	Map-RSeq	Transcriptome alignment, gene/exon count, fusion transcripts, single-nucleotide variant information	[46]
R	NOIseq	Determines noise within a dataset	[44]
R	sleuth	Observes low-abundance transcripts	[44]
R	DESeq2 v.1.20.0, count-per-million (EdgeR v.3.24.2), Linnorm v.2.6.0, SCnorm v.1.4.2, BASiCs v.1.4.0, scran v.1.8.2, TMM (EdgeR v.3.24.2)	Normalization of a dataset	[51]
R	Seurat v.2.3.4, Seurat3.0, RaceID3 v.0.1.3, RCA v.1.0, SC3 v.1.10.0, clusterExperiment v.2.2.0, MNN (scran v.1.8.2), CCA, scanorama v.1.0	Clustering analysis of a dataset	[51,52]
Python	BERMUDA, scVI	Clustering analysis of a dataset	[52]
R	DPT, Monocle2, Slingshot, SLICER, TSCAN	Trajectory analysis of a dataset	[51]
Python	NS-Forest v2.0, DESC	Machine learning of DEGs	[52,53]

**Table 3:** RNAseq methods applied to hPEC differentiation.

Cell Line	RNA Sequencing Method	Terminally Differentiated Cell Type	Reference
hESC H9	Integrated fluidic circuit scRNAseq	Retinal cells	[64]
Human bone-marrow-derived MSCs	Bulk RNAseq	Endothelial cells	[63]
iPSCs	Bulk RNAseq: KAPA Stranded mRNAseq Kit with BCL2FASTQ v1.8.4 software	Metanephric nephron progenitors, mesonephric nephron progenitors, ureteric bud	[69]
iPSCs	scRNAseq: ddSEQ with BCL2FASTQ v1.8.4 software	Metanephric nephron progenitors, mesonephric nephron progenitors, ureteric bud	[69]
iPSC	Genome-wide scRNAseq	Liver bud	[65,67]
hESC H9	Genome-wide scRNAseq	Pancreatic β-cells	[65]
Human embryo-derived developmental heart tissue	scRNAseq with CellRanger analysis with Seurat v2.3.4 software	Heart-derived cells: cardiomyocytes, endothelium, immune, epicardium, fibroblasts, smooth muscle, cardiac neural crest	[66]
NCRM-5 human iPSCs from male CD34^+^ cord blood	Bulk RNAseq with FASTQC, GENCODE v25, and STAR v2.5.2a software	Osteogenic progenitors	[68]
hESC H9	Bulk RNAseq with FASTQC, GENCODE v25, and STAR v2.5.2a software	Osteogenic progenitors	[68]

## Data Availability

The data supporting this review article are available in individual citations.
